# An exceptional horizontal gene transfer in plastids: gene replacement by a distant bacterial paralog and evidence that haptophyte and cryptophyte plastids are sisters

**DOI:** 10.1186/1741-7007-4-31

**Published:** 2006-09-06

**Authors:** Danny W Rice, Jeffrey D Palmer

**Affiliations:** 1 Department of Biology, Indiana University, Bloomington, IN 47405, USA

## Abstract

**Background:**

Horizontal gene transfer (HGT) to the plant mitochondrial genome has recently been shown to occur at a surprisingly high rate; however, little evidence has been found for HGT to the plastid genome, despite extensive sequencing. In this study, we analyzed all genes from sequenced plastid genomes to unearth any neglected cases of HGT and to obtain a measure of the overall extent of HGT to the plastid.

**Results:**

Although several genes gave strongly supported conflicting trees under certain conditions, we are confident of HGT in only a single case beyond the rubisco HGT already reported. Most of the conflicts involved near neighbors connected by long branches (e.g. red algae and their secondary hosts), where phylogenetic methods are prone to mislead. However, three genes – *clpP*, *ycf2*, and *rpl36 *– provided strong support for taxa moving far from their organismal position. Further taxon sampling of *clpP *and *ycf2 *resulted in rejection of HGT due to long-branch attraction and a serious error in the published plastid genome sequence of *Oenothera elata*, respectively. A single new case, a bacterial *rpl36 *gene transferred into the ancestor of the cryptophyte and haptophyte plastids, appears to be a true HGT event. Interestingly, this *rpl36 *gene is a distantly related paralog of the *rpl36 *type found in other plastids and most eubacteria. Moreover, the transferred gene has physically replaced the native *rpl36 *gene, yet flanking genes and intergenic regions show no sign of HGT. This suggests that gene replacement somehow occurred by recombination at the very ends of *rpl36*, without the level and length of similarity normally expected to support recombination.

**Conclusion:**

The *rpl36 *HGT discovered in this study is of considerable interest in terms of both molecular mechanism and phylogeny. The plastid acquisition of a bacterial *rpl36 *gene via HGT provides the first strong evidence for a sister-group relationship between haptophyte and cryptophyte plastids to the exclusion of heterokont and alveolate plastids. Moreover, the bacterial gene has replaced the native plastid *rpl36 *gene by an uncertain mechanism that appears inconsistent with existing models for the recombinational basis of gene conversion.

## Background

Unlike the dynamic mitochondrial genome of flowering plants, which frequently incorporates plastid and nuclear sequences via intracellular gene transfer [1-3], the plastid genome is highly resistant to the uptake of intracellular DNA [4,5]. Recently, a large number of discoveries of HGT involving mitochondrial genes of land plants have been reported [6-15]. Most, if not all of these transfers seem to be the result of a gene being transferred from the mitochondrial genome of one species to that of another. No analogous case of plastid-to-plastid transfer has been reported, but these mitochondrial discoveries recommend a thorough assessment of plastid HGT.

To date, only a single non-intron example of HGT to the plastid has been found. This is the ancient transfer of the rubisco operon (*rbcL *and *rbcS*) from a proteobacterium into the common ancestor of red algal plastids and their secondary derivatives [16], a case that is revisited in this study. In contrast to transfers of constituent genes, acquisition of new introns may be relatively common in plastids [17-25], based on their disparate phylogenetic distribution among plastid genomes, especially in green algae, and the fact that some introns are mobile elements.

The evidence found thus far for HGT to the plastid proceeded from studies of a particular gene or intron. To quantify the overall extent of HGT in plastid genomes, we searched exhaustively for HGT among the 42 sequenced plastid genomes available when this study began. Our search relied primarily on phylogenetic analyses, but also involved scrutiny of each potential case (including generation of new gene sequences from phylogenetically relevant taxa) to rule out artifacts and various types of homoplasy.

## Results

Of the 204 protein genes present in four or more of the 42 examined plastid genomes, 34 produced maximum likelihood (ML) trees that had at least one node that conflicted (see Methods) with the reference plastid tree (Additional File 1), with bootstrap proportion (BP) ≥ 80%. Fifteen had conflicts with BP ≥ 90%, and 11 had conflicts with BP ≥ 95%. Thirteen of the genes with BP ≥ 80% involved rhodophyte/*Odontella*/*Guillardia *relationships, eight involved conflicts within the four grass taxa, and the rest were various other conflicts. In eight trees, multiple nodes had well-supported conflicts. Usually these pointed to a single rearrangement in the tree, but three trees had well-supported conflicts in different regions of the tree.

After closer analysis, in some cases requiring the generation of additional sequences for key taxa, none of these conflicts proved to be strong cases of HGT. Ironically, one case that was not detected by this phylogenetic filter involves a very short gene that nonetheless offers strong support for a bacterium-to-plastid HGT.

### HGT of rpl36

**Figure 1:**
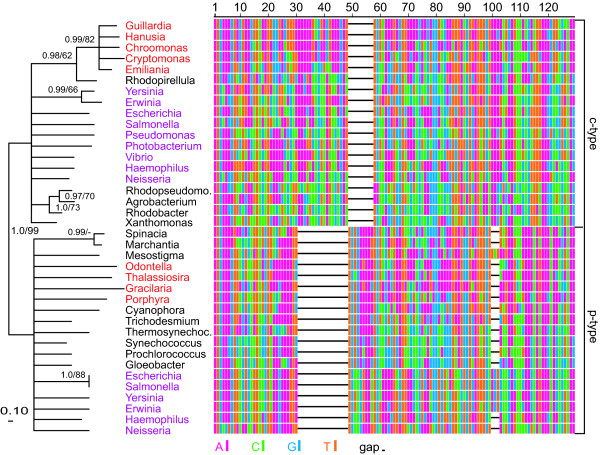
***rpl36 *tree and alignment**. The M3 codon model in MrBayes was used to calculate the tree using the alignment shown. Nodes with posterior probability <0.95 are collapsed. Posterior probabilities (left) and PROML BP values >50% (right) are shown on the remaining nodes. The PROML bootstraps were run with four rate categories (estimated with PUZZLE) and global rearrangements. Nucleotide and amino-acid based ML analyses using PAUP* and MrBayes also gave 100% support for the division between the c-type and p-type *rpl36 *genes. This support is maintained when all positions containing gaps are removed. Because the 3' extension unique to some c-type *rpl36 *genes (see Additional File 2) was excluded from this phylogenetic analysis, it is not shown in the alignment. In the alignment, each base is colored according to the key. Taxa in red include the red algae and their secondary plastid containing relatives. A subset of the many proteobacterial species which contain both the p-type and c-type genes is shown in purple. The p-type *Pseudomonas*, *Photobacterium*, and *Vibrio *genes are not shown here.

The *Guillardia theta rpl36 *gene is very divergent from the *rpl36 *genes present in the surveyed plastid genomes and in cyanobacteria. In trees, it branches with a paralogous *rpl36 *type with strong support regardless of the phylogenetic method used (Figure 1). Here we refer to the type found in *Guillardia *as *rpl36*-c (for cryptophyte), and the type found in most plastids and most cyanobacteria as *rpl36*-p (for plastid). The 144-bp-long *Guillardia *gene shares, with all *rpl36*-c genes relative to *rpl36*-p, three indels (insertions of one and six amino acids, and a deletion of three amino acids), as well as an overall amino-acid and nucleotide similarity (Figure 1 and Additional File 2). *Guillardia rpl36*-c has a 7 amino-acid 3' extension present in 18 gamma-proteobacterial species, in the planctomycete *Rhodopirellula baltica*, and in the cyanobacterium *Crocosphaera watsonii *(Additional File 2). The *rpl36 *HGT was not detected by our initial phylogenetic filter because our trees sampled only plastid-containing taxa, and this gene is too short to give strongly supported groupings within the plastids (Additional File 3). We detected this conflict only after building trees containing a broader sampling of *rpl36 *genes.

In addition to the plastids and cyanobacteria, *rpl36*-p is found across many groups of bacteria, including diverse proteobacteria, and in fungal nuclear genes targeted to the mitochondrion. Most gamma-proteobacteria and a few beta-proteobacteria and actinobacteria contain both forms of the *rpl36 *gene (e.g. Figure 1). *Crocosphaera watsonii *has an *rpl36*-p with a frame-shift insertion near the 5' end, suggesting that it has been functionally replaced by a horizontally transferred *rpl36*-c.

The *rpl36 *gene is located between *secY *and *rps13 *in all six sequenced plastid genomes from red algae and their secondary photosynthetic derivatives, including *Guillardia theta *(Additional File 2). This is within a larger syntenic group of 22 genes conserved in the red algal plastids and diverse bacterial lineages. None of the *rpl36*-c genes in bacteria are adjacent to *secY *or *rps13*, nor are any located within the larger syntenic region.

To identify the approximate time/phylogenetic boundary of transfer and to confirm the validity of the *Guillardia *gene, we sequenced *rpl36 *from three additional, diverse [26] cryptophytes: *Hanusia phi*, *Chroomonas mesostigmatica*, and *Cryptomonas tetrapyrenoidosa*. Using PCR, we isolated only *rpl36*-c from all three cryptophytes (and only *rpl36*-c was found in the unpublished plastid genome sequence of the cryptophyte *Rhodomonas salina *CCMP1319; H. Khan and J. Archibald, personal communication). These genes possess high sequence similarity (nucleotide identity between 79% and 96%) to *rpl36 *of *Guillardia theta*. We obtained high-quality sequence for a region comprising all of *rpl36*, both of its flanking spacers, 219 bp at the 3' end of *secY*, and 300 bp at the 5' end of *rps13 *(Figure 1 and Additional File 2).

The plastid genome of the haptophyte *Emiliania huxleyi*, which was sequenced too recently [27] to be included in this study, also contains *rpl36*-c in place of *rpl36*-p (Figure 1 and Additional File 2). *Emiliania rpl36 *shares the c-type indels and 3' extension with the cryptophyte *rpl36 *genes and contains no additional indels over its entire length. Its amino-acid identities to the cryptophyte *rpl36*-c genes range from 85 to 90%, and its nucleotide identities range from 72 to 79%. It too is located between *secY *and *rps13*, with 5' and 3' intergenic spacers of length 139 bp and 14 bp, respectively. The *Emiliania *sequence groups as sister to the cryptophyte *rpl36*-c genes with good support (Figure 1). In addition, an EST sequence  from the dinoflagellate *Karlodinium micrum*, which possesses a tertiary plastid of haptophyte origin [28-30], also contains the *rpl36*-c gene. Furthermore, the *Karlodinium *sequence is sister to the *Emiliania *sequence in phylogenetic analyses (data not shown, but see Additional File 2).

### The *clpP *conflict

In what follows, we describe and discuss genes that initially gave conflicting trees with relatively high bootstrap support, but which for various reasons were either strongly rejected or brought into question as potential horizontal transfers.

**Figure 2:**
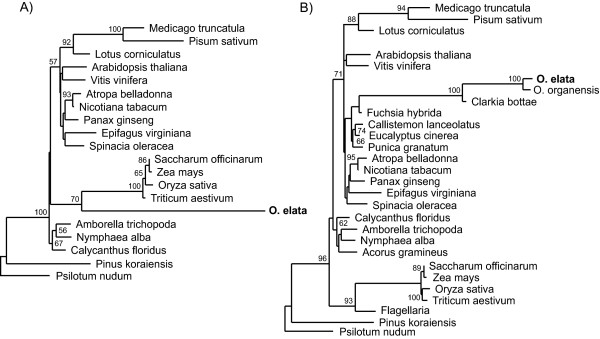
***clpP *phylogeny before and after taxon addition**. ML analysis was performed on an all-position nucleotide alignment using PAUP* as described in Methods with the TVM+G model used for both trees. A 60-bp 3' extension with questionable homology across taxa was removed in this analysis but was included in the original analysis. This is probably responsible for the change in the BP from 84% originally (not shown) to 70% here for *Oenothera *going with grasses in tree **(A)**. Bootstrap values <50% are not shown. **(A) **Original taxon sampling; **(B) **after new taxa added.

The *clpP *gene from *Oenothera elata *is highly divergent. With the taxon sampling used in this study (Additional File 1), it branches, with 84% BP (see Figure 2 legend), as the sister to the grasses, which are also a long-branched group (Figure 2A). Suspecting long-branch attraction (LBA), we obtained more genes (provided by L. Goertzen and C. Long) from the order Myrtales, to which *Oenothera *belongs (including *Clarkia*, *Fuschia*, *Eucalyptus*, *Punica*, *Callistemon*, and *Oenothera organensis*) and from other non-grass monocots (including *Acorus *and *Flagellaria*) to test if the grouping with the grasses was an artifact. The resulting tree (Figure 2B) strongly suggests that the original result was an LBA artifact, as *Oenothera *goes within the Myrtales (and within its family Onagraceae) with the better sampling.

### The *ycf2 *conflict

**Figure 3:**
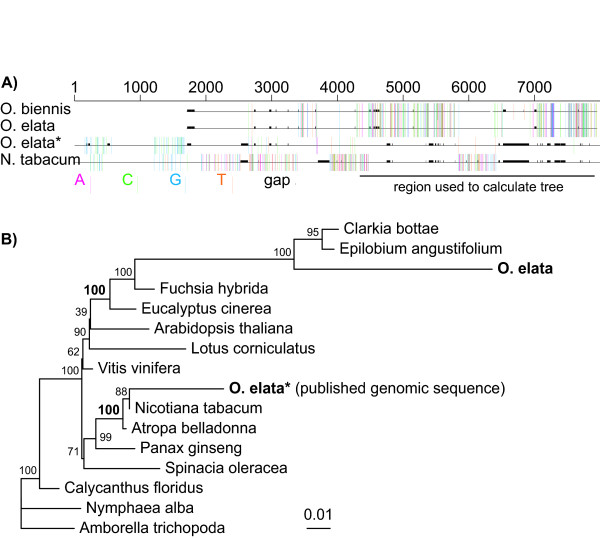
**Error in the published *Oenothera elata ycf2***. **(A) **Alignment of *ycf2 *nucleotide sequences: The top two sequences, *Oenothera biennis *and *O. elata*, were sequenced as part of this study. We did not sequence the first ~1600 bp. The bottom two sequences correspond to the published *Oenothera elata *and *Nicotiana tabacum *sequences. The bottom three sequences were used to determine a consensus base at each position, and positions that did not match this consensus are colored as denoted in the key. **(B) **All codon position ML tree using the TVM+G model in PAUP* with 100 bootstrap replicates. Only the 3' region of *ycf2 *starting at position 4023 of the published *Oenothera *sequence [31] was obtained for the four Myrtales taxa (*Eucalyptus*, *Fuchsia*, *Clarkia *and *Epilobium*) and the analysis was performed using this region of aligned positions as indicated in **(A)**. Within this region, gappy positions were removed prior to phylogenetic analysis, which resulted in 2567 positions. When the entire gene was used with the published *Oenothera *sequence excluded, the topology was the same except that the *Lotus *and *Arabidopsis *branches were switched. When the published *Oenothera *is included in the full-length analysis, its strong chimerism pulled *Clarkia*, *Epilobium *and our *elata *sequence into an artifactual clade with the published *elata *gene at the base of the Solanaceae.

The published *Oenothera elata ycf2 *gene branched as sister to the asterids *Atropa *and *Nicotiana *with BP of 100%, instead of with other rosid sequences (e.g. *Arabidopsis *and *Lotus*). To verify this, we sequenced *ycf2 *from a number of diverse Myrtales including *O. elata *itself. This new *Oenothera *sequence did not match the published *O. elata *plastid genome sequence [31], several regions of which (up to 1.5 kb in length) have 100% sequence identity with the *Nicotiana ycf2 *gene (Figure 3A). These cover regions that have long insertions in our *O. elata *sequence but that are missing in the *O. elata *genome sequence. This latter sequence also contains insertions shared with *Nicotiana *but not with our *O. elata ycf2 *sequence. Regions in the published sequence that do match our sequence appear to have single base errors, given that our *O. biennis *sequence is more similar to our *O. elata *sequence than is the published sequence in these regions (Figure 3A). Although very divergent, the new *O. elata *sequence branches in the expected position with other Myrtales (Figure 3B). We strongly suspect that this conflict is due to extensive error in the *ycf2 *region of the published *O. elata *genome [31], perhaps due to inadvertent incorporation of *Nicotiana *sequence during genome assembly (see Additional File 4).

### The rhodophyte/chromalveolate conflicts

The chromalveolates [*Guillardia *(cryptophytes), *Odontella *(heterokonts), and apicomplexans in our sampling] are a putatively monophyletic group that, with respect to plastid phylogeny, branch within the red algae, with the Cyanidiales being sister to the *Porphyra*/*Gracilaria*/chromalveolate clade (Additional File 1). Relationships among the different chromalveolate groups are not well established [32], but the consensus topology provides a reasonable working hypothesis. Most of the conflicts that we found relative to this topology involve *Guillardia *and/or *Odontella *branching as sister to all the rhodophytes instead of as sister to *Porphyra*/*Gracilaria*. Although some of these conflicts could in principle be true cases of HGT, the combination of long branches and near-neighbor exchanges makes these conflicts suspect, even given high bootstrap support. For some of these we have seen evidence (assuming our plastid tree is correct) of codon-usage bias. For example, the *psbB *gene tree goes from 100% BP for rhodophytes being monophyletic to the exclusion of *Guillardia *and *Odontella*, using all three codon positions, to weak support for a topological change to the consensus tree when only second positions are used. First and second positions together still give strong support for the conflicting tree, indicating that first positions may also contribute a significant bias for *psbB *[33].

Another well-supported conflict, psbA, is discussed in Additional File 5. The remaining genes that had conflicts supported by BP of 80% or higher are *psbC*, *atpH*, *psaK*, *dnaK*, *atpF*, *atpB*, *rpl31*, *ycf4*, *ycf17*, *ycf45*, and *ycf37*. As above, we could not reject the conflicts outright, but we could show weakened support or induce topological changes with alternative data filtering such as using second codon positions alone or amino acids. In no case did we observe any telltale signals such as uniquely shared indels in the conflicting clades.

### The grass conflicts

Eight gene trees conflicted with the consensus tree (Additional File 1) with respect to relationships among the four grasses examined. Four gene trees (*atpI*, *psbH*, *atpF*, *rpl16*) supported the monophyly of *Triticum*/*Saccharum*/*Zea*, while the other four (*rpl22*, *ndhD*, *psaA*, *psbA*) supported monophyly of *Oryza*/*Saccharum*/*Zea*. In all cases there is a long branch leading to the grasses, which reflects both the lack of any close outgroups (no other monocots were included) and the well-established rapid evolution of the chloroplast genome in the stem group leading to grasses (Stefanovic et al [34] and references therein). We hypothesize that all eight gene trees whose within-grass topologies conflicted with the consensus topology reflect spurious results stemming from the lack of close outgroups to the grass sequences. To test this hypothesis, we reanalyzed the two genes (*atpI *and *psbH*) that showed the highest level of conflict (BP = 96%), using all other monocot sequences available (from *Sorghum, Hordeum, Phyllostachys, Typha, Yucca, Phalaenopsis, and Acorus*) for these two genes. With this improved sample, the *atpI *phylogeny was no longer in significant conflict with the organismal tree; instead, relationships among *Triticum*, *Oryza*, and *Saccharum/Zea *were entirely unresolved under an all nucleotide position ML model (Additional File 6). For *psbH*, however, the situation did not change markedly; we obtained a BP of 91% for *Oryza *being sister to *Triticum*/*Saccharum*/*Zea*, but this corresponds to only three parsimony informative characters. Such a small number of informative characters could easily be homoplasious, and better taxon sampling is required to resolve these conflicts firmly.

### Other conflicts

The remaining conflicts with BP ≥ 80% were all brought into question using alternative phylogenetic analyses that led to reduction of bootstrap support or topology change. All of these involved conflicts with branches that are near each other in the consensus tree.

### rRNA and tRNA genes

The small and large subunit ribosomal RNA genes had strong support for the euglenids going outside the green algae (sister to red/green algae for 16S and within the red algae for 23S). Increasing taxon sampling using other available rRNA genes [35] gave a weakly supported placement of the euglenids within the green algae. No clear cases of HGT in tRNA genes were detected, but interpretation of these alignments and trees is difficult, owing to the short length and extensive paralogy of these genes.

### Scrutiny of long-branched lineages

The approach used here is limited by the taxon sampling. Because our initial trees included only plastid genes, we could essentially detect HGT only from one plastid genome to another, but not transfers from other genomes into plastids. We reasoned that transfers of non-plastid genes should normally result in a long branch leading to the donee taxon within the plastid gene trees. A description of our analysis of these long-branch lineages is presented in Additional File 7. No additional cases of HGT were detected in this analysis.

## Discussion

### The *rpl36 *transfer: the chromalveolate hypothesis and algal phylogeny

The unique, derived presence of the horizontally transferred *rpl36*-c gene in haptophyte and cryptophyte plastids, but not in heterokont and alveolate plastids, provides the first strong evidence for the "sisterhood" of haptophyte and cryptophyte plastids. The most parsimonious scenario is that the *rpl36*-c gene was transferred once to the ancestral plastid of the haptophytes and cryptophytes after this plastid lineage and the lineage(s) leading to heterokonts and alveolates diverged. Less parsimonious alternative scenarios can be imagined, but the similarity between the haptophyte and cryptophyte *rpl36 *genes, their position as sister lineages among the *rpl36*-c genes (Figure 1), and the fact that the transfer appears to have occurred via an improbable recombination event make alternative explanations unlikely.

The haptophytes and heterokonts have been recognized as sister groups based on ultrastructural and pigment similarities [36,37], and named 'chromobiotes'. In addition, phylogenies based on concatenated plastid genes tend to group the haptophytes and heterokonts [38,39] (see Additional File 8 for further discussion of chromalveolate phylogeny). However, on a per-gene basis, the signal is mixed, and nearly half the plastid genes actually group haptophytes and cryptophytes as sisters (Additional File 9 and Additional File 10). The morphological characters linking haptophytes to heterokonts could all be ancestral to the chromophytes (chromobiotes plus cryptophytes) or even the chromalveolates (chromophytes plus alveolates) [32,40]) and lost differentially. For example, chlorophyll c3 and autofluorescence of the rear cilium could have been lost in the cryptophytes, and the nucleomorph (present in cryptophytes) could have been lost independently in the haptophytes and heterokonts (it is well established that the nucleomorph has been independently lost in the secondary, green-derived plastid of euglenids). In contrast, the presence of the c-type *rpl36 *in only haptophytes and cryptophytes cannot be explained by differential loss unless one posits its unlikely insertion via HGT immediately adjacent to, rather than in place of, the ancestral p-type gene. It remains to be seen whether the hypothesis of haptophyte/cryptophyte plastid monophyly is supported or rejected by future phylogenetic analyses involving many more plastid and nuclear genes and/or taxa from across the chromalveolates. One possibility, which is based on the serial symbiosis models developed by Bachvaroff et al [38,39], is that the cryptophyte and haptophyte plastids, but not their nuclear lineages, will turn out to be sister groups. This would be the case if, say, the cryptophyte plastid was of secondary, red-algal origin and the haptophyte plastid of tertiary, cryptophyte origin. However, a study using six nuclear cytosolic protein genes did group haptophytes and cryptophytes with weak support [41], suggesting that their nuclear genes may also be monophyletic.

### The donor of the *rpl36*-c gene

*rpl36*-c was probably transferred to the ancestral plastid of haptophytes and cryptophytes directly from a bacterium rather than from the mitochondrion or nucleus, as there is no evidence of a *rpl36*-c in these compartments. At the amino-acid level, plastid *rpl36*-c is most similar to *Rhodopirellula baltica*. Interestingly, the complete shotgun sequences from two other planctomycetes (*Blastopirellula marina *and *Gemmata obscuriglobus*) contain only *rpl36*-p. Thus, a potential transfer between the *Rhodopirellula *lineage and the cryptophyte lineage would likely postdate the *Rhodopirellula*/*Blastopirellula*/*Gemmata *divergence. However, a recent HGT from an unknown donor into *Rhodopirellula *is also possible. On balance, based on the current bacterial sampling, the donor of the plastid *rpl36*-c was most likely a planctomycete related to *Rhodopirellula *or a proteobacterium. A cyanobacterium related to *Crocosphaera watsonii *is a less likely but potential donor since the *Crocosphaera *branches within the gamma-proteobacterial c-type group (tree not shown; but see Additional File 2), but was probably recently acquired from this group via HGT (see Results).

### The *rpl36 *transfer: mechanism and functional consequence

Because plastid *rpl36*-c and *rpl36*-p are both located between *secY *and *rps13 *in the same orientation, we suspect that the *rpl36 *HGT was mediated by homologous recombination. This would be extraordinary, because the *Guillardia *and *Porphyra rpl36 *genes are only 49% identical in nucleotide sequence in non-gap regions. At this level of divergence, homologous recombination is thought to be highly unlikely [42,43]. It is implausible that flanking sequence could have been used to initiate gene conversion, as intergenic regions between distant taxa are essentially random, and no bacterial c-type *rpl36 *genes are flanked by *secY *and *rps13*. Additionally, the 3' end of *secY *and the 5' end of *rps13 *in *Guillardia *do not appear to have been replaced by divergent sequence, as they are still highly similar to the red algal and cyanobacterial genes relative to *Rhodopirellula *and proteobacteria (Additional File 2A). Even the last 30 bases of *Guillardia secY *have a higher sequence identity to red algal and cyanobacterial homologs than to all other known sequences. As the 3' end of the *rps13 *first and second position alignment is iteratively removed, *Guillardia *continues to group with the red algae and cyanobacteria until about 40 bp are left, at which point phylogenetic resolution is lost, owing to relatively high sequence conservation. There is no significant sequence similarity between the *Guillardia rpl36 *intergenic regions and those from any available c-type-containing bacteria. In fact, there is only modest conservation among the intergenic regions of the additional cryptophyte genomes we examined. The cryptophyte intergenic spacers 3' to *rpl36 *ranged in length from 42 to 53 bp with sequence identities, in non-gap regions, of 68–80%, while the 5' spacers ranged in length from 30 to 150 bp with sequence identities of 59–74%.

This leads to the hypothesis that recombination may have been initiated by very short regions of conservation between the *rpl36*-c and *rpl36*-p genes themselves. Most reported recombination events between bacterial species tend to be among highly similar sequences [44]. However, this may not be entirely due to the level of sequence similarity but also to interspecies barriers, such as mismatch repair [45]. The minimal sequence identity required to initiate recombination varies depending on the system and species being tested, but has been shown to involve 20 or fewer consecutive identical nucleotides for some types of recombination [46,47].

Although the overall similarity between *rpl36*-c and *rpl36*-p is very low (Figure 1), the 5' and 3' ends are more conserved than the rest of the gene. Specifically, the plastid *rpl36*-c genes share an 8-bp 5' sequence (AGTAAAGT) with all *rpl36*-p genes in the red algal lineage, as well as with several green algae and land plants and with several *rpl36*-p and *rpl36*-c bacterial genes. A less well-conserved 6-bp 3' sequence (CAAGGT) exists in cryptophyte and some alpha-proteobacterial *rpl36*-c genes and in the *rpl36*-p gene from some cyanobacteria, land plants, green algae, and red algae. This identity extends leftwards by a further 3 bp (CGTCAAGGT) in *Cryptomonas*, some cyanobacteria, and *Odontella*. These similarities between the plastidal and bacterial *rpl36*-c and the *rpl36*-p genes in the red-plastid lineage are consistent with one or both ends being involved in recombination. A gene replacement along these lines would represent, to our knowledge, an unprecedented recombination event in terms of sequence distance. Although it is conceivable that the *rpl36*-c and *rpl36*-p genes involved in this putative recombination some 1 billion years ago shared more sequence similarity than the extant genes, *rpl36*-c has not diverged greatly among haptophytes and cryptophytes, and *rpl36*-p is quite conserved among plastids (Figure 1).

This being the case, possible alternatives to recombination, and HGT itself, should be considered, such as convergent evolution. However, even though *rpl36 *is very short, convergence is unlikely, given the sequence divergence between the p- and c-type *rpl36 *genes and that the algal c-type genes emerge as a nested clade from within the larger group of c-type genes (Figure 1). The convergence hypothesis would require a staggering and unprecedented number of convergent events. At the amino-acid level, the *Guillardia rpl36 *shares 36 identities with *Rhodopirellula*, but only 13–18 identities with its red-algal relatives. It also shares three gaps and a 7 amino-acid 3' extension with bacterial *rpl36*-c (Figure 1 and Additional File 2). Although functional convergence does occur in protein genes [48], nothing approaching the extent that must be invoked for *rpl36 *has been shown. Protein functional convergence usually occurs at very short, key areas of the protein; for example, within active site regions of an enzyme.

In contrast to convergent positive selection for function, GC content can have a dramatic effect on amino-acid and codon usage [49]. However, plastids, including *Guillardia *and *Emiliania*, have a low genomic GC content relative to the bacterial genomes of the taxa shown in Figure 1. The *rpl36*-c genes mirror the genomic GC content. For example, *Guillardia rpl36*-c is 31% GC (genome is 33%) while *Rhodopirellula rpl36*-c is 54% (genome is 55%). In contrast to convergence, these differences in GC content probably account for much of the divergence between the plastid and bacterial *rpl36*-c genes and make chance convergence much less likely.

*rpl36*-c almost certainly physically replaced *rpl36*-p in certain algae; but did it also functionally replace it? The two types are highly divergent from one another (Figure 1 and Additional File 2), and to our knowledge, *rpl36*-c has never been shown to play the equivalent ribosomal function in any organism. It is therefore conceivable that a nuclear-encoded, plastid-targeted *rpl36*-p exists in the cryptophytes and haptophytes, while *rpl36*-c serves some other function. However, upon consideration of the crystal structure of the 50S ribosomal subunit from *E. coli *[50] it is plausible that *rpl36*-c could functionally replace *rpl36*-p. First, the amino acids making van der Waals and hydrogen bond contacts with the 23S ribosomal RNA are fairly well conserved between the two *rpl36 *types (Additional File 2). Second, the region where the 3 amino-acid insertion exists in the c-type makes no intermolecular contacts, but instead protrudes into the solvent where an insertion is unlikely to cause a functional problem. Interestingly, this insertion creates potential N-myristoylation and N-glycosylation sites that could have functional importance. Third, the crystal structure reveals a large empty space, in contact with the C-terminal glycine, that could easily accommodate the 7 amino-acid C-terminal extension in *rpl36*-c. Thus, it is reasonable to expect that *rpl36*-c could functionally replace *rpl36*-p without any major steric interference or loss of intermolecular contacts. In addition, *rpl36*-c is highly conserved between the haptophyte and cryptophytes as would be expected for a functional ribosomal protein.

### Rubisco HGT revisited

It was first recognized nearly 20 years ago [51-53] that red algae and their secondary symbiotic derivatives possess a rubisco operon (*rbcLS*) of highly unusual evolutionary origin. Whereas all green plastids and those of glaucophytes contain *rbcLS *of expected cyanobacterial origin, red plastids possess rubisco genes of apparent proteobacterial origin. Based on phylogenetic considerations, Delwiche and Palmer [16] argued in 1996 that the red algal rubisco was acquired from proteobacteria by horizontal transfer in the common ancestor of all red algae. In addition, they provided evidence for several other *rbcLS *transfers, all among eubacteria. Martin and Schnarrenberger [53] argued that the cyanobacterial endosymbiont instead carried both the red-like and green-like rubisco genes from a duplication predating cyanobacteria and proteobacteria, and that differential loss in the plastid lineages and loss in all cyanobacterial lineages resulted in the observed pattern.

**Figure 4:**
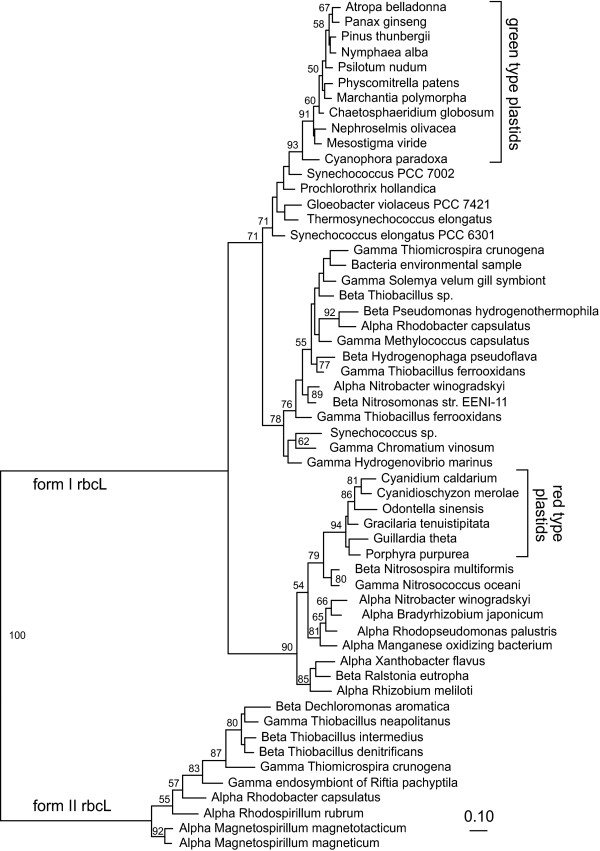
**Phylogenetic tree of red-like and green-like *rbcL *sequences**. The amino-acid Bayesian tree was generated using MrBayes with the following parameters: *rates *= invgamma; *aamodelpr *= mixed; *ngen *= 500000; *nchains *= 4. The *burnin *was set to 100 to generate the tree and this *burnin *gave a convergence diagnostic of 0.017. The nodal support values are PROML bootstrap support values obtained using global rearrangements, and four rate categories and an invariant category estimated using PUZZLE. Support values are shown on nodes with BP ≥ 50.

In the context of the current study, and with the passage of some 10 years and the accumulation of hundreds of bacterial genome sequences, we revisit this issue. Figure 4 shows an *rbcL *phylogeny for a representative sampling of currently available sequences. The overall structure of this tree is very similar to that of Figure 2 of the paper by Delwiche and Palmer [16]. Importantly, however, a number of new proteobacterial *rbcL *sequences have become available that show even greater similarity to red algal *rbcL *than those considered by that study [16]. For example, the recently sequenced genome of *Nitrosospira multiformis *shares 86% amino-acid identity with *Gracilaria *over a contiguous 381 amino-acid region of *rbcL*. This is within the range of identities among the red algae over this same region. The *rbcL *tree (Figure 4) groups the red algal *rbcL *genes with those of *Nitrosospira *and *Nitrosococcus*. With the advent of these and other related bacterial sequences, the red algal *rbcL *clade is now two steps nested within the overall clade of red-like proteobacterial *rbcL *sequences, whereas previously [16] it was simply sister to a more limited set of proteobacteria.

Now, with many more rubisco sequences in hand, and with complete genomes available for many of these organisms, the duplication/loss model strongly conflicts with the phylogenetic and presence/absence data. None of the 15 or more sequenced cyanobacterial genomes contains a red-like *rbcL*. Furthermore, out of the many bacterial *rbcL *sequences now available, only a single organism, *Rhodobacter azotoformans*, has been found that contains both red-like and green-like *rbcL *[54], and this is clearly due to a bacterial HGT event instead of retention of both copies from an ancient duplication. So the hypothesis of an ancient duplication and differential loss of paralogs is becoming increasingly untenable.

Alternatively, instead of a transfer to the recent ancestor of the red algae, a recent ancestor of the cyanobacterial endosymbiont could have received a red-like *rbcL *from a proteobacterium followed by differential losses in the plastid lineages [55]. This possibility is less parsimonious, however, as it still requires one horizontal transfer, plus at least two independent losses in the plastid lineage and at least one in cyanobacteria. In conclusion, it is likely that the *rbcLS *operon of red algae represents a genuine HGT event to the plastid genome.

### HGT in plastids: rare but choice

Comprehensive examination of all 204 genes present in four or more of the 42 examined plastid genomes has revealed but a single new, well-supported case of HGT. This *rpl36 *transfer and the *rbcL*S transfer described years ago [16] and revisited above share several features: (i) they both involve bacterial donors; (ii) they are both relatively ancient [56-58], having occurred in the common ancestor of red algae (*rbcLS*), perhaps 1.0–1.5 billion years ago, or in the common ancestor of cryptophytes and haptophytes (*rpl36*), probably not much more recently; and (iii) in both cases, the transferred genes are known (*rbcLS*; see Delwiche and Palmer [16,51-53], Valentin and Zetche [16,51-53], Boczar et al [16,51-53], Martin and Schnarrenberger [16,51-53], and references therein) or thought (*rpl36*) to have functionally replaced native homologs, which would explain their retention for eons as intact genes. The *rpl36 *transfer also serves as an important phylogenetic marker and is intriguing from a mechanistic standpoint. Thus, although HGT in plastids is extremely rare, when it happens it can be of considerable consequence and interest.

Both cases of plastid horizontal gene transfer occurred anciently in red algae or their secondary derivatives, while several cases of potential [22] or likely [20,21] horizontal acquisition of introns are evident in green algae plastids. In contrast, no cases of HGT were evident in our analyses of sequenced plastid genomes of land plants, nor has HGT been reported for any of the many plastid genes that have been widely sequenced (in hundreds to thousands of plants) for phylogenetic purposes. This contrast is noteworthy because far more plastid sequencing has been performed in land plants (99671 entries from an NCBI Entrez search for plastid genes) than in algae (6731 entries). Are algal plastids, an admittedly paraphyletic group, somehow more amenable to HGT than plant plastids?

### HGT and getting the right tree

We initially constructed many phylogenetic trees that gave well-supported, conflicting results suggestive of HGT, but which were ultimately deemed wrong or showed weakened support under closer scrutiny. The largest source of conflicts arose within the red algal lineage and their secondary descendants (and to a lesser extent with the green algae), where limited taxon sampling and early diversification of lineages led to a series of long terminal branches connected by short internal branches. This is where phylogenetic reconstruction is most prone to fail due to LBA [59]. Subsequent analyses with improved taxon sampling and/or filtering of fast-evolving codon positions caused us to reject all of these cases as potential HGTs. Some of these conflicts might still represent actual HGT events, but further taxon sampling will be required to resolve the issue completely. Sequencing errors should be considered a possibility and the anomalous sequences verified where appropriate.

### Why is HGT so less common in plastids than in mitochondria in land plants?

Some 40 cases of HGT have now been reported in plant mitochondrial genomes [6-15] versus none in plant plastids. This is despite far less sequencing of plant mitochondrial genes (7075 Genbank entries) than plastid genes (99671 Genbank entries). Similarly, plant mitochondrial genomes are rich in plastid and nuclear sequences acquired via intracellular gene transfer [1-3], whereas plastid genomes entirely lack such sequences [4,5]. What could account for such differences? To be sure, plant mitochondrial genomes are less compact (72–89% noncoding DNA in sequenced angiosperm genomes) and less constrained in size (varying over 10-fold in size). Nevertheless, angiosperm plastids contain considerable noncoding DNA (generally 40–45%), suggesting that the greater propensity for mitochondrial HGT is not simply a function of the total amount of "junk" DNA. Rather, the differences may be how efficiently the organelles take up exogenous DNA. Plant mitochondria possess an active DNA uptake system [2]; no similar activity has been reported for plastids, but it is also unclear whether this has been assayed for. This uptake system may lower a rate-limiting barrier in the incorporation of both foreign and native DNA. A major, well-documented difference between the two organelles is the tendency of mitochondria to fuse. This may account for some of the observed mitochondrion-to-mitochondrion HGT. Plant mitochondria regularly fuse [60,61], promoting recombination between parental mitochondrial genomes in the case of somatic hybrid plants generated by protoplast fusion, whereas chloroplasts virtually never fuse under similar conditions [62,63].

## Conclusion

This study confirms and quantifies the hypothesis that HGT is rare in plastids. Only *rpl36 *and the rubisco operon are clear cases of HGT to the plastid genome. Both are ancient transfers, whereby bacterial genes have replaced native homologs and have become permanent, functional residents in their respective lineages. In contrast, the frequent (and recent) transfers in plant mitochondria occur by plant-to-plant transfer and are essentially ephemeral events, few of which seem to be of functional significance. The horizontal transfer of bacterial *rpl36*-c into the plastid genome represents an unprecedented example of apparent homologous recombination that defies current concepts of the sequence relatedness required to allow gene conversion/replacement to occur. The *rpl36*-c HGT also serves as a striking phylogenetic character that establishes an important new phylogenetic connection, linking haptophyte and cryptophyte plastids as sister groups to the exclusion of heterokont and alveolate plastids.

## Methods

### Plastid genomes

EMBL-Bank files for the following 40 plastid genomes were retrieved from the European Bioinformatics Institute: *Eimeria tenella *(AY217738), *Euglena gracilis *(X70810), *Euglena longa *(AJ294725), *Guillardia theta *(AF041468), *Toxoplasma gondii *(U87145), *Cyanophora paradoxa *(U30821), *Cyanidioschyzon merolae *(AB002583), *Cyanidium caldarium *(AF022186), *Gracilaria tenuistipitata *(AY673996), *Porphyra purpurea *(U38804), *Odontella sinensis *(Z67753), *Adiantum capillus *(AY178864), *Amborella trichopoda *(AJ506156), *Anthoceros formosae *(AB086179), *Arabidopsis thaliana *(AP000423), *Atropa belladonna *(AJ316582), *Calycanthus floridus *(AJ428413), *Chaetosphaeridium globosum *(AF494278), *Chlamydomonas reinhardtii *(BK000554), *Chlorella vulgaris *(AB001684), *Epifagus virginiana *(M81884), *Lotus japonicus *(AP002983), *Marchantia polymorpha *(X04465), *Medicago truncatula *(AC093544), *Mesostigma viride *(AF166114), *Nephroselmis olivacea *(AF137379), *Nicotiana tabacum *(Z00044), *Nymphaea alba *(AJ627251), *Oenothera elata *(AJ271079), *Oryza sativa *(X15901), *Physcomitrella patens *(AP005672), *Pinus koraiensis *(AY228468), *Pinus thunbergii *(D17510), *Psilotum nudum *(AP004638), *Saccharum officinarum *(AP006714), *Spinacia oleracea *(AJ400848), *Triticum aestivum *(AB042240), *Zea mays *(X86563), *Plasmodium falciparum *(X95275, X95276), *Panax ginseng *(AY582139). In addition, the sequence of the *Pisum sativum *plastid genome was provided by John C. Gray (unpublished data), and the *Vitis vinifera *coding sequences were extracted and pieced together (unpublished result) from all NCBI nucleotide databases including dbEST [64]. A combination of BLAST searches with closely related genomes, Perl scripts for parsing output, and hand editing was used to define the protein and RNA genes in the unannotated genomes of *Pisum sativum*, *Vitis vinifera*, and *Medicago truncatula*.

### Gene clustering

In total, 5676 protein, tRNA and rRNA genes were extracted from the 42 plastid genome sequences. A BLAST [65] database was created with these DNA sequences, and then each sequence was used as a BLAST query against the database. From the BLAST output, a pairwise distance matrix was constructed based on the best BLAST expectation value for each query/hit pair. For a pair to be considered, the BLAST expectation value had to be ≤0.1, and at least 20% of the longer sequence of a pair had to be included in the alignment. Pairs for which these criteria were not met received a large distance value of 1.1. A huge neighbor-joining tree was constructed with PAUP* software, [66] using this distance matrix. Gene families and superfamilies were easily identifiable in the resulting tree. Unrelated gene families formed a polytomy of long branches at the root node. From this tree, 204 protein gene families containing four or more genes were hand selected by visual inspection. Ribosomal RNA genes were easily resolved in the tree, but transfer RNA genes were clustered into many hard-to-resolve paralogous families. The distinct tRNA clusters were separated into groups for further clustering using maximum parsimony (MP) and ML analyses.

### Gene alignment

Protein and nucleotide alignments were made for each of the gene families using MUSCLE software [67] with unlimited iterations, and were inspected manually to correct errors. Initially, amino-acid alignments were constructed for sequences whose translation could be obtained. The protein alignment was then back-translated to nucleotides using the known nucleotide sequences. Sequences that could not be translated (such as pseudogenes and RNA genes) were aligned based on nucleotides. Positions containing mostly gaps, especially where homology was deemed questionable, were excluded from phylogenetic analyses.

### Phylogenetic analyses

ML models for each gene family were determined using the likelihood-ratio test criterion of MODELTEST [68] except where specified. Final model parameter values were estimated by iteratively building ML trees and recalculating parameter values until the best trees converged. Heuristic ML searches using tree bisection-reconnection branch swapping in PAUP* were performed to find these trees. All three codon positions were included for the initial phylogenetic screening of gene families, but first and second position and other character-sampling strategies and software were used secondarily if needed to clarify the phylogenetic support for a conflicting tree. One hundred ML bootstrap replicates were performed using the same model and search method as used for searches for the best trees. Neighbor-joining and MP analyses were also carried out to allow for comparison to the ML results. Additional phylogenetic methods and programs (e.g. MrBayes, PAML and PROML) are indicated where used.

### Phylogenetic conflict evaluation

A consensus plastid tree (Additional File 1) was used as the working hypothesis topology for finding conflicts in gene trees. This tree was compiled from the current literature and our unpublished work using entire genomes. Nodes in a gene tree that conflicted with the plastid tree by addition of a taxon not part of that clade or subtraction of a taxon that is part of that clade were marked as in conflict. Conflicting nodes were ordered by their ML BP values, using a PERL script developed for finding these conflicts. Trees were viewed graphically, with conflicting clades highlighted to determine whether further processing was necessary. To rule out HGT in well-supported conflicts, we scrutinized the alignment in more detail, increased taxon sampling, and tried other models and phylogenetic methods.

### New gene sequences

Several new sequences for *rpl36 *(DQ365944–DQ365946) and *ycf2 *(DQ370441–DQ370447) were obtained using standard PCR and sequencing protocols [69]. Cryptophyte genomic DNAs (for *rpl36 *isolation) were generously provided by Chris Lane and John Archibald. Angiosperm DNAs (for *ycf2 *isolation) were isolated [70] directly from young plant leaves or were taken from lab stocks.

## Authors' contributions

DWR carried out all the analyses, wrote the new software used in this study, generated the new *rpl36 *and *ycf2 *sequence data, and drafted the manuscript (text and figures). DWR and JDP jointly directed the project, and JDP contributed substantially to the final manuscript. Both authors read and approved the final manuscript.

## Supplementary Material

Additional file 1 Consensus plastid phylogeny. Shown is the plastid phylogeny used in this study, based on the current literature. Dashed and solid vertical brackets denote paraphyletic and monophyletic groups, respectively. Although the tree is shown as entirely resolved, some parts are not well supported (e.g., relationships among *Cyanophora*, green algae, and red algae; among bryophytes; among chromalveolates; and whether *Amborella *plus *Nymphaea *or *Amborella *alone is the sister of all other angiosperms). Click here for file

Additional file 2 Amino-acid alignment of *secY*, *rpl36 *and *rps13 *and the rp*l*36 amino-acid alignment showing the C-terminal extensions and atomic contacts in the ribosome crystal structure. **(A) **This shows that *secY *and *rps13 *from plastids which contain the c-type *rpl36 *are more similar to *secY *and *rps13 *genes from red algae and cyanobacteria than to potential *rpl36 *donors, *Rhodopirellula *and proteobacteria. Amino acids that conflict with the consensus amino acid at each position are colored according to the key. The similarity between the c-type *rpl36 *genes in the haptophyte and cryptophyte plastids and those from *Rhodopirellula *and proteobacteria is also apparent. Note that *rpl36 *is flanked by *secY *and *rps13 *in plastids of red algal origin, but the c-type *rpl36 *in bacteria is not flanked by these genes. **(B) **Amino-acid alignment of c-type and p-type *rpl36 *genes. Note the three apicomplexans included in the alignment (*Plasmodium*, *Toxoplasma*, and *Theileria*). At the bottom is shown which residues make contact with the 23S rRNA in the ribosome crystal structure of *Escherichia coli*, which has a p-type *rpl36*. Click here for file

Additional file 3 The poorly resolved *rpl36 *tree obtained in the initial phylogenetic screen. This shows the *Guillardia rpl36 *gene going arbitrarily with *Pisum *with weak support given the poor taxon sampling and low information content of this gene. See Figure 1 for clarification. Click here for file

Additional file 4 Discussion of the *Oenothera elata ycf2 *gene error in the genome sequence. Discussion of the *Oenothera elata ycf2 *gene error in the genome sequence. Click here for file

Additional file 5 Results of the *psbA *conflict in the red algal lineage. Results of the *psbA *conflict in the red algal lineage. Click here for file

Additional file 6 Resolution of conflicts within grasses by adding taxa for the *atpI *gene. **(A) **The topology and support values with the original taxon sampling. The conflicting node is indicated by the bold 96; **(B) **after more monocot taxa are added *Triticum *and *Zea *move apart. Click here for file

Additional file 7 List of gene trees having branches ≥ 4 SD above the mean branch length for a given tree and text describing analysis of long branch taxa. The text describes the analysis of long-branch taxa. The table gives the number of standard deviations above the mean, the corresponding gene and the group that the long-branch leads to. Click here for file

Additional file 8 Discussion of chromalveolate hypothesis and algal phylogeny. Discussion of chromalveolate hypothesis and algal phylogeny. Click here for file

Additional file 9 Multigene chromist plastid phylogeny. Results of the phylogenetic placement analysis of the haptophyte *Emiliania *based on all plastid genes. Click here for file

Additional file 10 Plastid genes supporting the sisterhood of either haptophytes and heterokonts or haptophytes and cryptophytes. The histograms list the genes favoring the given topology over the other according to the codeml amino-acid ML score with the WAG matrix [71] and gamma distributed rates. The site-specific likelihood values were calculated using parameter estimates and branch lengths based on the concatenated alignment with all the genes. The height of the histogram bars represent the log likelihood preference for the given topology over the other for a particular gene (i.e. the sum of site likelihoods in the concatenated alignment corresponding to a particular gene). **(A) **The topology found using MrBayes with 21,659 plastid amino-acid positions shared among the chromophytes, *Gracilaria*, and *Porphyra*. Invariant sites and gamma distributed rates were used with the Cprev model [72]. The posterior probabilities were 1.0 for all nodes. **(B) **The topology was found as in **(A) **except that *Guillardia *and *Emiliania *were constrained to be monophyletic. The node corresponding to the chromist clade had a posterior probability of 0.98 and the other nodes had 1.0. Click here for file
