# Supplementary figures and images for: An exceptional horizontal gene transfer in plastids: gene replacement by a distant bacterial paralog and evidence that haptophyte and cryptophyte plastids are sisters
Source: BMC Biol. 2006 Sep 6;4:31. doi: 10.1186/1741-7007-4-31 (PMC1570145; doi:10.1186/1741-7007-4-31)

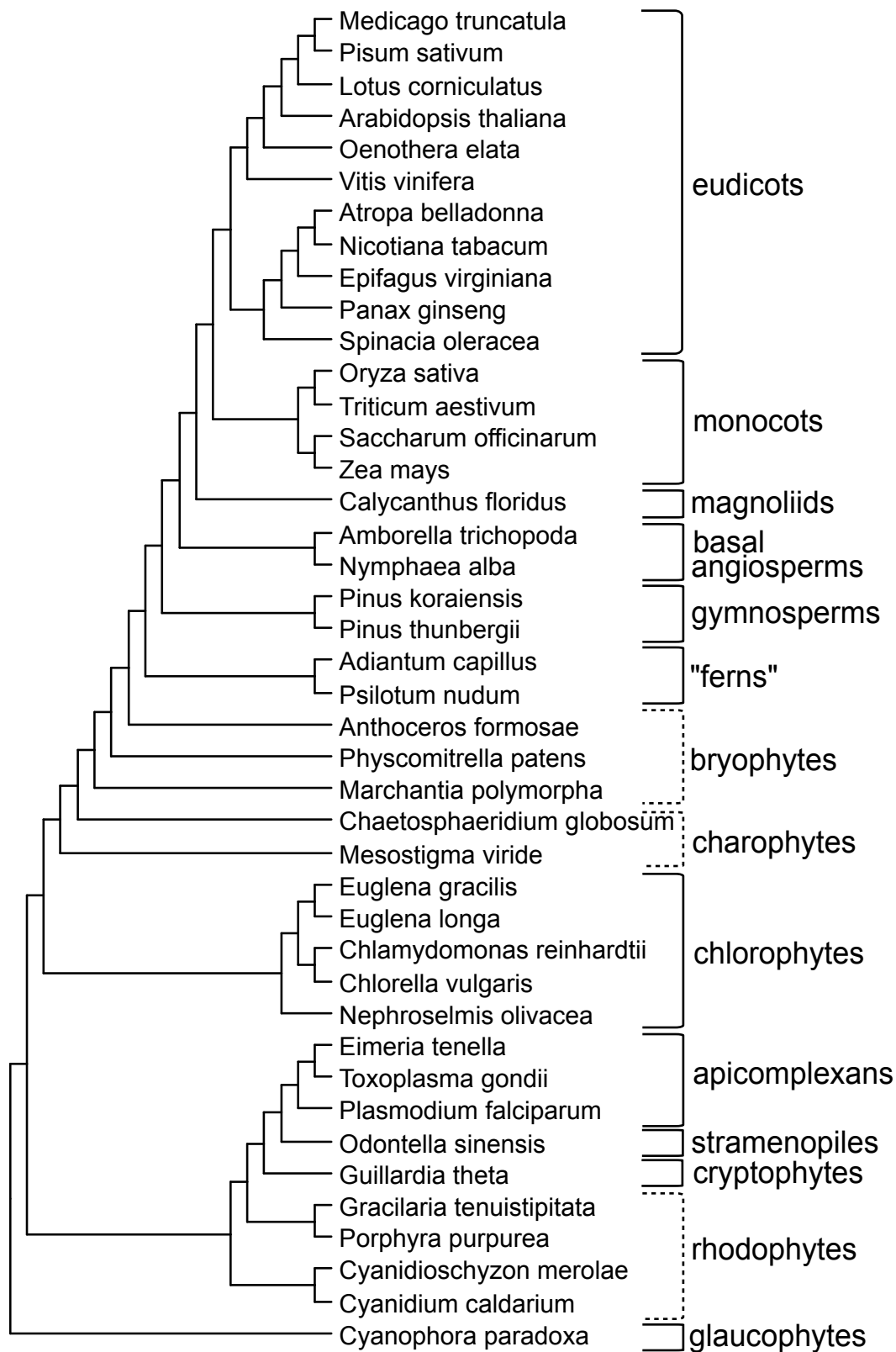

Supplement: Additional file 1 — Consensus plastid phylogeny. Shown is the plastid phylogeny used in this study, based on the current literature. Dashed and solid vertical brackets denote paraphyletic and monophyletic groups, respectively. Although the tree is shown as entirely resolved, some parts are not well supported (e.g., relationships among Cyanophora, green algae, and red algae; among bryophytes; among chromalveolates; and whether Amborella plus Nymphaea or Amborella alone is the sister of all other angiosperms). [file 1741-7007-4-31-S1.pdf]

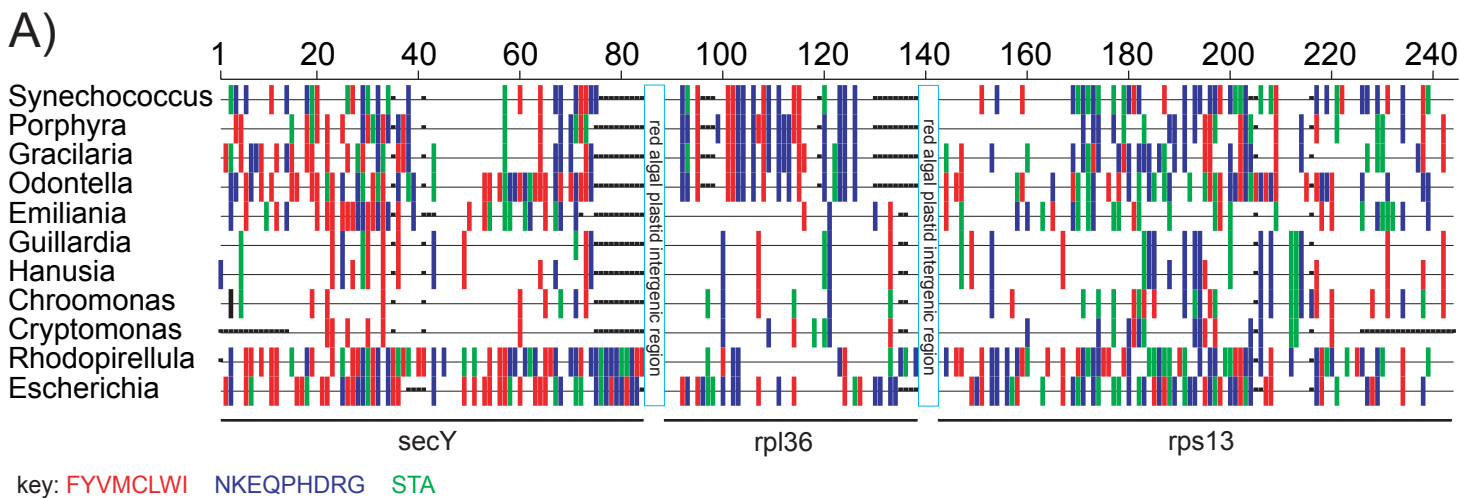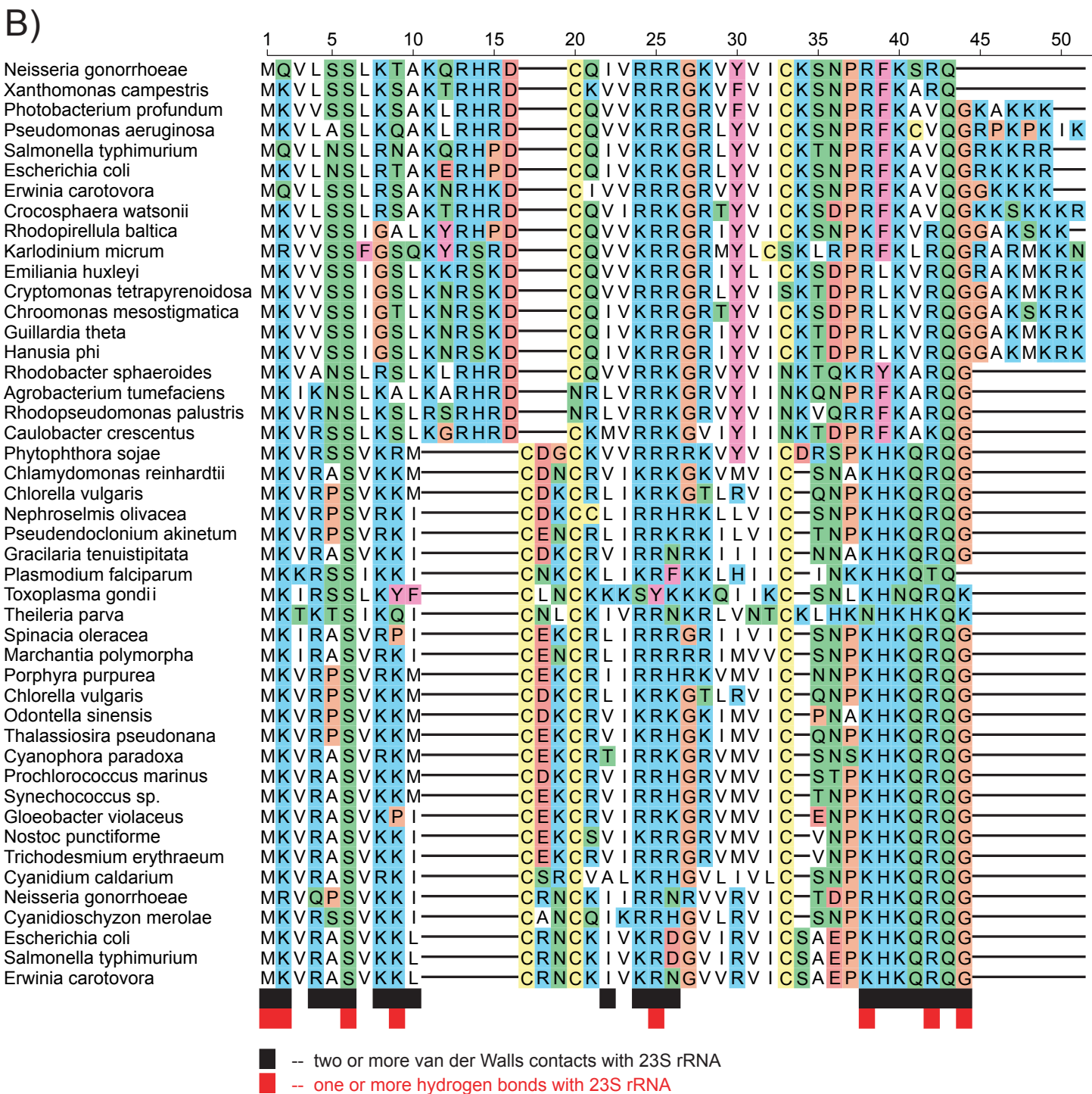

Supplement: Additional file 2 — Amino-acid alignment of secY, rpl36 and rps13 and the rpl36 amino-acid alignment showing the C-terminal extensions and atomic contacts in the ribosome crystal structure. (A) This shows that secY and rps13 from plastids which contain the c-type rpl36 are more similar to secY and rps13 genes from red algae and cyanobacteria than to potential rpl36 donors, Rhodopirellula and proteobacteria. Amino acids that conflict with the consensus amino acid at each position are colored according to the key. The similarity between the c-type rpl36 genes in the haptophyte and cryptophyte plastids and those from Rhodopirellula and proteobacteria is also apparent. Note that rpl36 is flanked by secY and rps13 in plastids of red algal origin, but the c-type rpl36 in bacteria is not flanked by these genes. (B) Amino-acid alignment of c-type and p-type rpl36 genes. Note the three apicomplexans included in the alignment (Plasmodium, Toxoplasma, and Theileria). At the bottom is shown which residues make contact with the 23S rRNA in the ribosome crystal structure of Escherichia coli, which has a p-type rpl36. [file 1741-7007-4-31-S2.pdf]

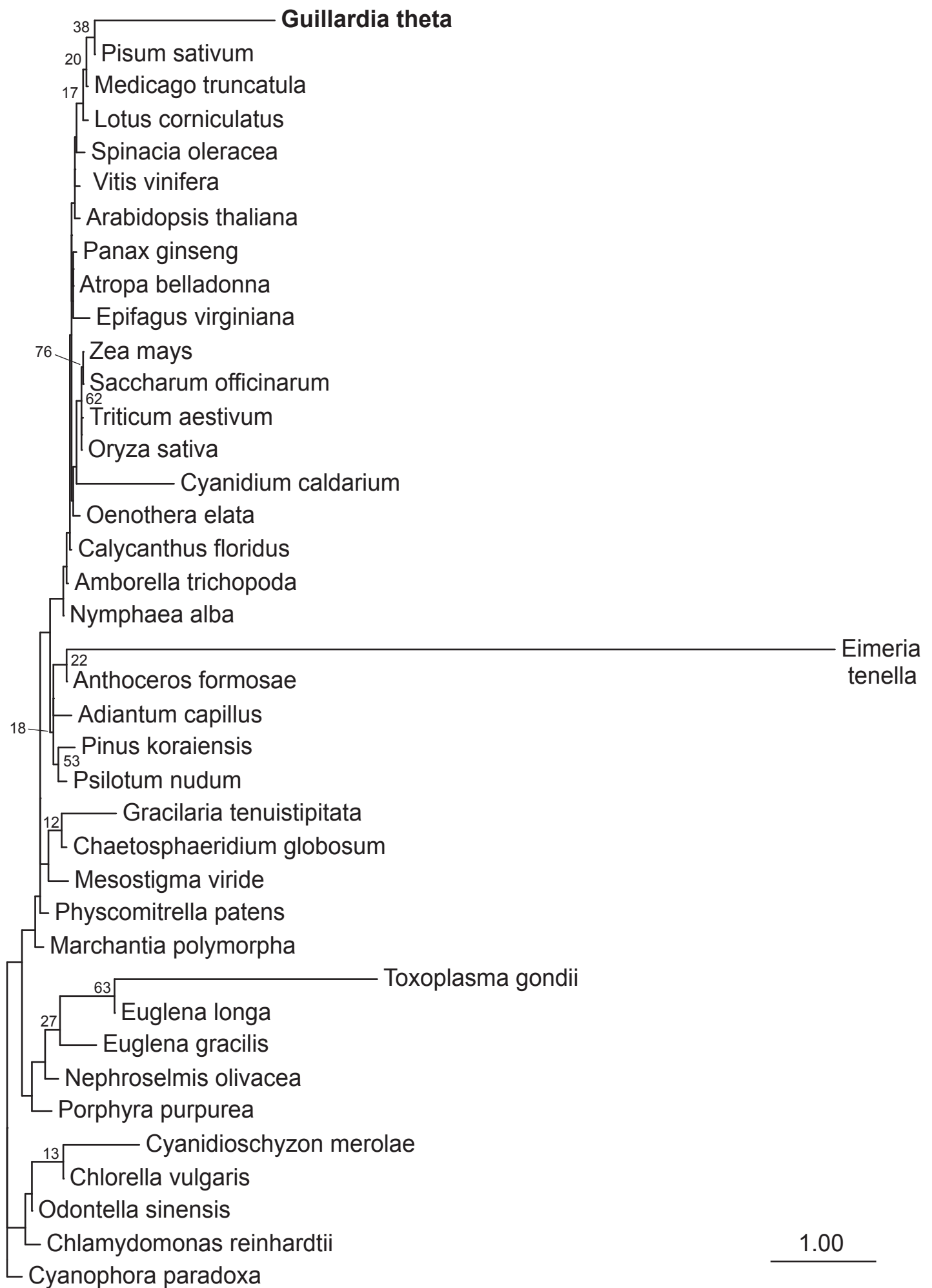

Supplement: Additional file 3 — The poorly resolved rpl36 tree obtained in the initial phylogenetic screen. This shows the Guillardia rpl36 gene going arbitrarily with Pisum with weak support given the poor taxon sampling and low information content of this gene. See Figure 1 for clarification. [file 1741-7007-4-31-S3.pdf]

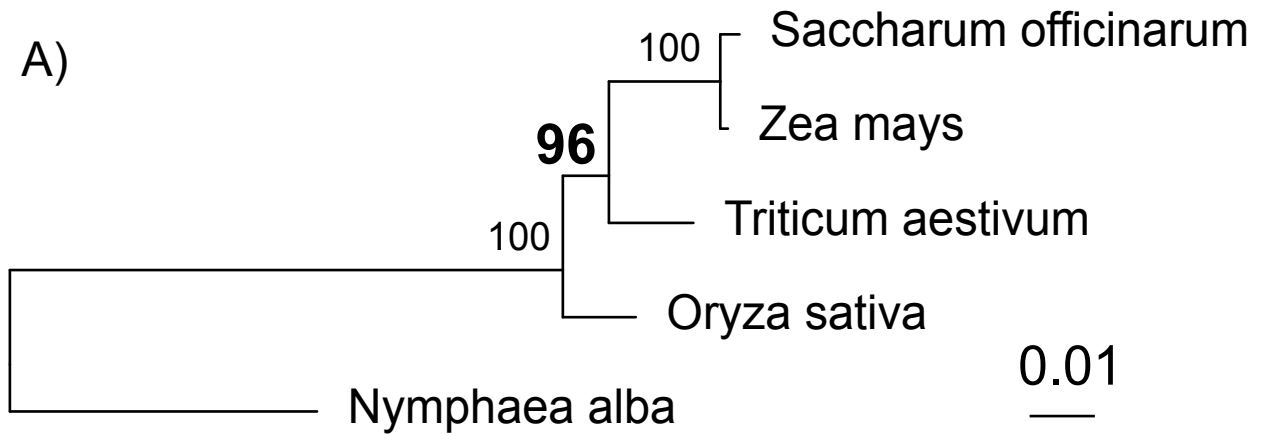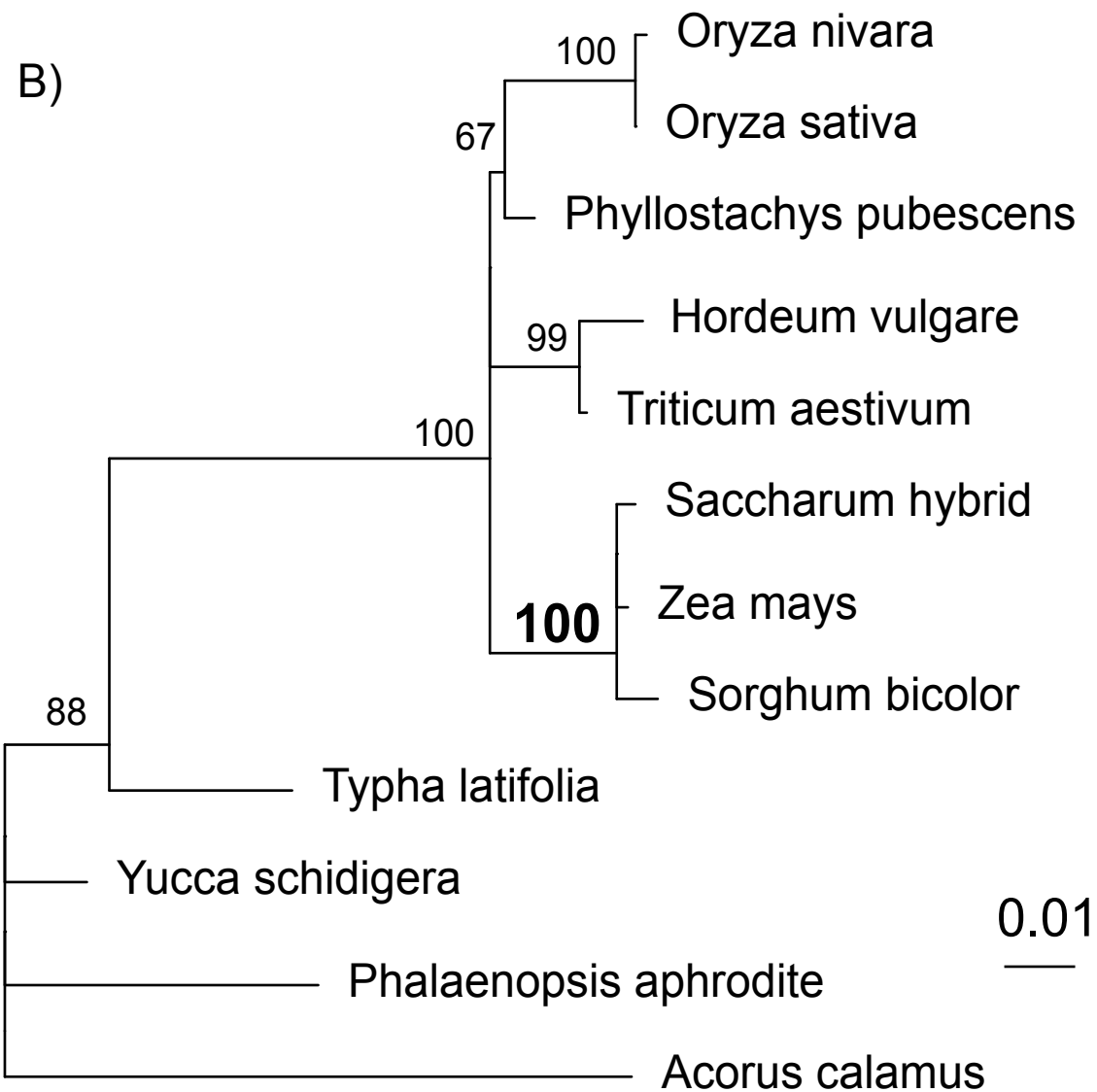

Supplement: Additional file 6 — Resolution of conflicts within grasses by adding taxa for the atpI gene. (A) The topology and support values with the original taxon sampling. The conflicting node is indicated by the bold 96; (B) after more monocot taxa are added Triticum and Zea move apart. [file 1741-7007-4-31-S6.pdf]

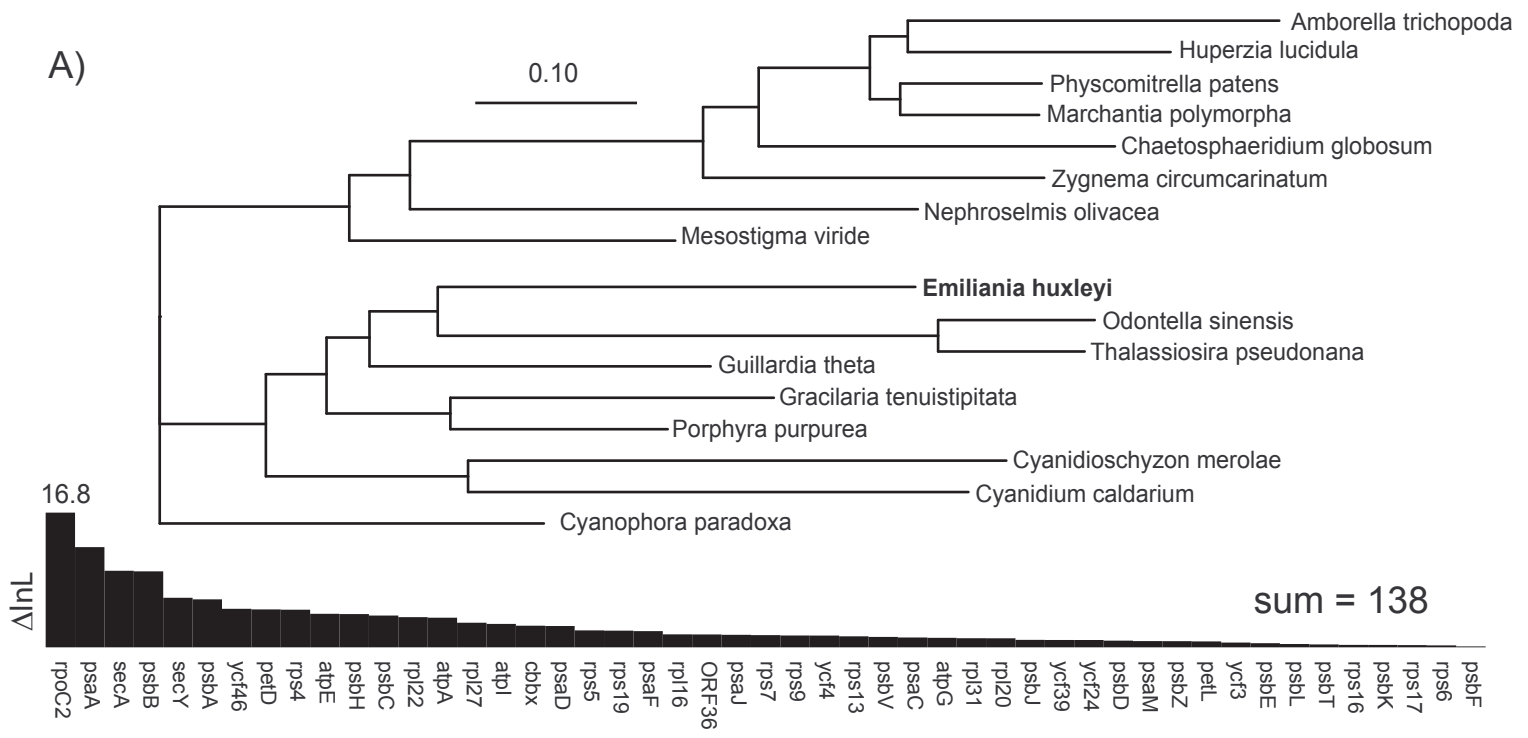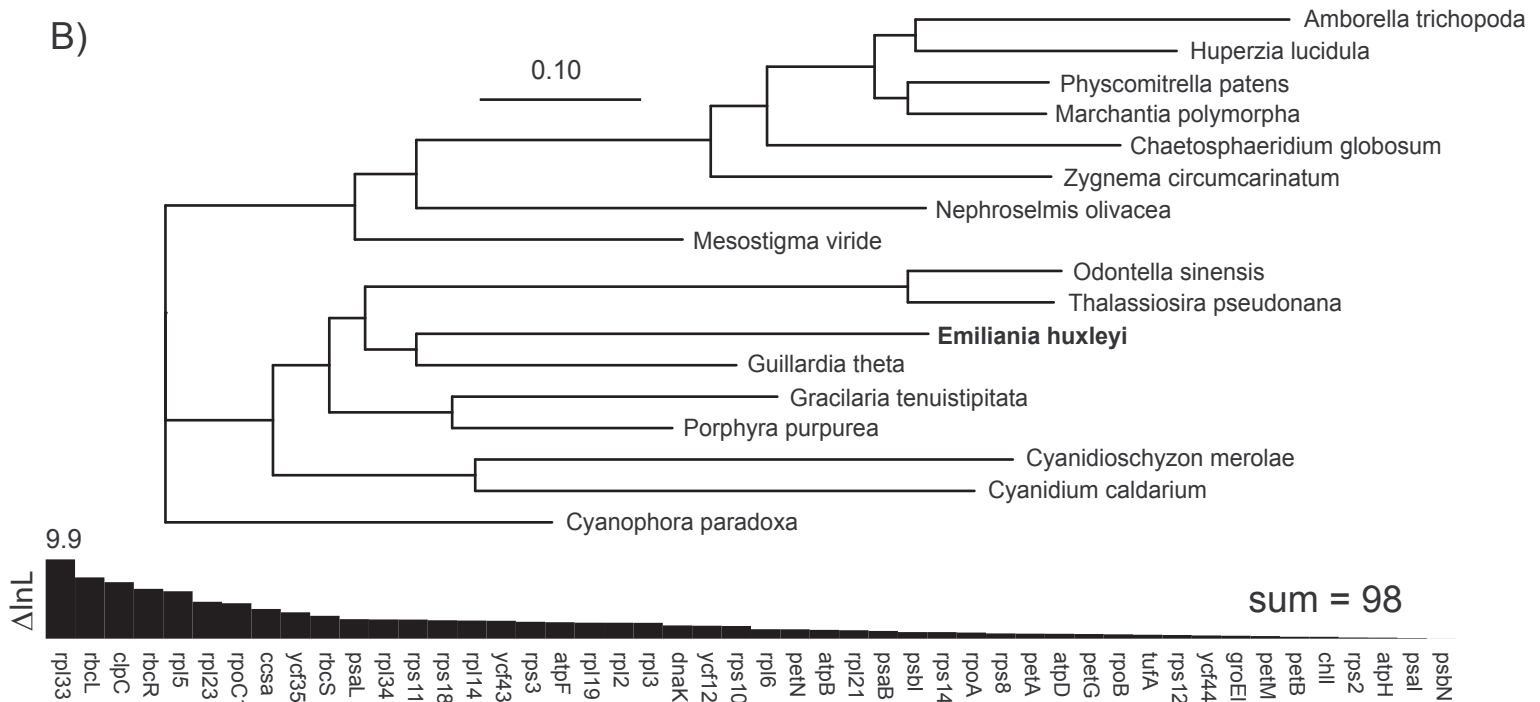

Supplement: Additional file 10 — Plastid genes supporting the sisterhood of either haptophytes and heterokonts or haptophytes and cryptophytes. The histograms list the genes favoring the given topology over the other according to the codeml amino-acid ML score with the WAG matrix [71] and gamma distributed rates. The site-specific likelihood values were calculated using parameter estimates and branch lengths based on the concatenated alignment with all the genes. The height of the histogram bars represent the log likelihood preference for the given topology over the other for a particular gene (i.e. the sum of site likelihoods in the concatenated alignment corresponding to a particular gene). (A) The topology found using MrBayes with 21,659 plastid amino-acid positions shared among the chromophytes, Gracilaria, and Porphyra. Invariant sites and gamma distributed rates were used with the Cprev model [72]. The posterior probabilities were 1.0 for all nodes. (B) The topology was found as in (A) except that Guillardia and Emiliania were constrained to be monophyletic. The node corresponding to the chromist clade had a posterior probability of 0.98 and the other nodes had 1.0. [file 1741-7007-4-31-S10.pdf]
