# Supplementary material for: An exceptional horizontal gene transfer in plastids: gene replacement by a distant bacterial paralog and evidence that haptophyte and cryptophyte plastids are sisters
Source: BMC Biol. 2006 Sep 6;4:31. doi: 10.1186/1741-7007-4-31 (PMC1570145; doi:10.1186/1741-7007-4-31)
Supplement: Additional file 4 — Discussion of the Oenothera elata ycf2 gene error in the genome sequence. Discussion of the Oenothera elata ycf2 gene error in the genome sequence. [file 1741-7007-4-31-S4.pdf]

ycf2 error in the Oenothera elata genome

The sequencing results show that extensive regions of the 7 kb *ycf2* locus from *Nicotiana tabacum* (tobacco), the very first angiosperm plastid genome to be sequenced [1], were somehow incorporated into the *O. elata* genome sequence when it was assembled and published [2]. A previously sequenced 345-bp repeat region of the *Oenothera elata ycf2* gene [3] agrees with our sequence exactly but differs from the genome sequence, providing independent validation of our sequence. Alternatively, but extremely unlikely, a very recent HGT event occurred between *Nicotiana* and the *O. elata* strain used for genome sequencing by Hupfer et al. [2]. With this likely error in mind, we inspected the rest of the published genome sequence for *O. elata* and did not find any other strong evidence for tobacco sequences erroneously incorporated into the genome sequence (there may be a small amount of tobacco sequence incorporated in the *O. elata* 23S rRNA gene, but we did not pursue this further). Nonetheless, the published plastid genome sequence of *O. elata* should be treated with caution in all phylogenetic and other comparative studies.

1. Shinozaki K, Ohme M, Tanaka M, Wakasugi T, Hayashida N, Matsubayashi T, Zaita N, Chunwongse J, Obokata J, Yamaguchi-Shinozaki K, et al: **The complete nucleotide sequence of the tobacco chloroplast genome: its gene organization and expression.** *EMBO J* 1986, **5:**2043-2049.
2. Hupfer H, Swiatek M, Hornung S, Herrmann RG, Maier RM, Chiu WL, Sears B: **Complete nucleotide sequence of the *Oenothera elata* plastid chromosome, representing plastome I of the five distinguishable euoenothera plastomes.** *Mol Gen Genet* 2000, **263:**581-585.
3. Blasko K, Kaplan SA, Higgins KG, Wolfson R, Sears BB: **Variation in copy number of a 24-base pair tandem repeat in the chloroplast DNA of *Oenothera hookeri* strain Johansen.** *Curr Genet* 1988, **14:**287-292.
