# Supplementary material for: An exceptional horizontal gene transfer in plastids: gene replacement by a distant bacterial paralog and evidence that haptophyte and cryptophyte plastids are sisters
Source: BMC Biol. 2006 Sep 6;4:31. doi: 10.1186/1741-7007-4-31 (PMC1570145; doi:10.1186/1741-7007-4-31)
Supplement: Additional file 5 — Results of the psbA conflict in the red algal lineage. Results of the psbA conflict in the red algal lineage. [file 1741-7007-4-31-S5.pdf]

Rhodophyte/Chromalveolate conflicts - psbA

As another example, the *psbA* tree puts *Guillardia* as sister to *Porphyra*/*Gracilaria* with BP = 99% to the exclusion of *Odontella*, which branches with the Cyanidiales with weak support. Using the reasonably good taxon sampling available for *psbA* [1, 2], in analyses with either 2nd positions alone or all positions, the heterokonts and haptophytes go with the Cyanidiales while the cryptophytes go with the other rhodophytes (data not shown). However, the bootstrap support is reduced to below 80% for relevant nodes. Surprisingly, nucleotide MP, which is more susceptible to LBA, recovers (with weak support) the standard topology when all three codon positions are used. Ironically, though, 2nd codon position MP reverts to the tree obtained with ML. This gene has been noted previously to be problematic [1, 3], perhaps owing to unusual evolutionary pressures operating on its protein product, or, less likely, a history of HGT.

1. Bachvaroff TR, Sanchez-Puerta MV, Delwiche CF: **Chlorophyll c-containing plastid relationships based on analyses of a multigene data set with all four chromalveolate lineages.** *Mol Biol Evol* 2005, **22:**1772-1782.
2. Yoon HS, Hackett JD, Pinto G, Bhattacharya D: **The single, ancient origin of chromist plastids.** *Proc Natl Acad Sci U S A* 2002, **99:**15507-15512.
3. Inagaki Y, Simpson AGB, Dacks JB, Roger AJ: **Phylogenetic artifacts can be caused by leucine, serine, and arginine codon usage heterogeneity: Dinoflagellate plastid origins as a case study.** *Syst Biol* 2004, **53:**582-593.
