# Supplementary material for: An exceptional horizontal gene transfer in plastids: gene replacement by a distant bacterial paralog and evidence that haptophyte and cryptophyte plastids are sisters
Source: BMC Biol. 2006 Sep 6;4:31. doi: 10.1186/1741-7007-4-31 (PMC1570145; doi:10.1186/1741-7007-4-31)
Supplement: Additional file 7 — List of gene trees having branches ≥ 4 SD above the mean branch length for a given tree and text describing analysis of long branch taxa. The text describes the analysis of long-branch taxa. The table gives the number of standard deviations above the mean, the corresponding gene and the group that the long-branch leads to. [file 1741-7007-4-31-S7.pdf]

Scrutiny of long branched lineages

The approach employed here is limited by the taxon sampling. Because our initial trees included only plastid genes, we could essentially detect HGT only from one plastid genome to another, but not transfers from other genomes into plastids. For example, the well-known *rbcL* conflict was not detected in our initial automated analyses because the *rbcL* tree was rooted on *Cyanophora* and the red algae came out artifactually in their expected position (albeit with a very long branch). In the case of *rpl36*, *Guillardia* branched within angiosperms, but with very weak support (Additional File 3). If the *Guillardia* gene were longer we probably would have recognized this conflict. However, unless a conflict arising from a transfer outside of plastids fortuitously results in the standard plastid topology (as for the red algae *rbcL*), or the bootstrap support is low, such conflicts would be detected by our initial phylogenetic screen.

To detect any other *rbcL*-like situations, we parsed all of our gene trees to find branches significantly longer than the average branch length for that tree. We calculated the z-score for each branch in each tree as $z_i = (bl_i - \overline{bl})/sd$ where bl_i is a particular branch length in a tree, $\overline{bl}$ is the mean branch length for that tree, and sd is the standard deviation of branch lengths for that tree. The z-scores for the branches leading to the red algae for *rbcL* and to *Guillardia* for *rpl36* were among the highest, with z-scores greater than 6.0 (see table below). Many of the large z-scores were from the highly divergent and AT-rich genes of the apicomplexans, as expected [1-3]. Excluding branches leading to apicomplexans from the calculation, we analyzed the 45 genes with z-scores greater than 4.0 by searching the protein sequences of the long branch taxa against all available bacterial and eukaryotic nucleotide sequences in the default NCBI database using TBLASTN and building trees from the taxa hit by BLAST [4]. None of these showed the level of phylogenetic support for extra-plastidic HGT found for *rbcL* and *rpl36*, and none were deemed good candidates. Although many genes were very divergent from all other plastid genes, none had unambiguous affinity to other, non-plastid groups.

z-score	gene	taxon
**7.84**	**rbcL**	**Red lineage**
7.65	petN	Pinus koraiensis
6.87	rpl23	Oryza sativa
6.83	rps2	Chlamydomonas reinhardtii
**6.45**	**rpl36**	**Guillardia theta**
6.21	psbG	Pinus koraiensis
5.88	psbN	Euglena gracilis
5.56	psbA	Euglena gracilis
5.54	rpl14	Euglena longa
5.40	rps12	Euglena longa
5.13	ndhA	Nephroselmis olivacea
5.10	psbB	Euglena gracilis
5.07	petG	Cyanidium caldarium
5.06	psbJ	Chlamydomonas reinhardtii
4.96	ycf4	Euglena gracilis
4.93	matK	Chaetosphaeridium globosum
4.88	psbK	Euglena gracilis
4.80	rpoA	Euglenids
4.78	tufA	Chaetosphaeridium globosum

4.77	ndhH	Nephroselmis olivacea
4.77	atpH	Euglena gracilis
4.72	psbC	Euglena gracilis
4.69	ndhJ	Adiantum capillus
4.68	ycf9	Cyanidium caldarium
4.64	ycf2	Chaetosphaeridium globosum
4.60	rpl22	Cyanidium caldarium
4.60	psbI	Euglena gracilis
4.55	psbT	Nephroselmis olivacea
4.54	psaA	Euglena gracilis
4.52	rps18	Cyanidium caldarium
4.52	psbD	Euglena gracilis
4.51	rps3	Euglena longa
4.49	rps11	Cyanidium caldarium
4.44	rpoC2	Chlorella vulgaris
4.39	rpl2	Cyanidioschyzon merolae
4.35	ycf9	Euglena gracilis
4.21	ndhG	Nephroselmis olivacea
4.15	psaJ	Cyanophora paradoxa
4.07	rps7	Euglena longa
4.07	rpl20	Euglenids
4.04	rpl32	Cyanidioschyzon merolae
4.03	rps15	Adiantum capillus
4.00	atpE	Euglena gracilis

1. Wilson RJ, Denny PW, Preiser PR, Rangachari K, Roberts K, Roy A, Whyte A, Strath M, Moore DJ, Moore PW, Williamson DH: **Complete gene map of the plastid-like DNA of the malaria parasite *Plasmodium falciparum*.** *J Mol Biol* 1996, **261:**155-172.
2. Kohler S, Delwiche CF, Denny PW, Tilney LG, Webster P, Wilson RJ, Palmer JD, Roos DS: **A plastid of probable green algal origin in Apicomplexan parasites.** *Science* 1997, **275:**1485-1489.
3. Cai X, Fuller AL, McDougald LR, Zhu G: **Apicoplast genome of the coccidian *Eimeria tenella*.** *Gene* 2003, **321:**39-46.
4. Altschul SF, Madden TL, Schaffer AA, Zhang J, Zhang Z, Miller W, Lipman DJ: **Gapped BLAST and PSI-BLAST: a new generation of protein database search programs.** *Nucleic Acids Res* 1997, **25:**3389-3402.
