# Supplementary material for: An exceptional horizontal gene transfer in plastids: gene replacement by a distant bacterial paralog and evidence that haptophyte and cryptophyte plastids are sisters
Source: BMC Biol. 2006 Sep 6;4:31. doi: 10.1186/1741-7007-4-31 (PMC1570145; doi:10.1186/1741-7007-4-31)
Supplement: Additional file 8 — Discussion of chromalveolate hypothesis and algal phylogeny. Discussion of chromalveolate hypothesis and algal phylogeny. [file 1741-7007-4-31-S8.pdf]

Previous phylogenetic studies of chromalveolates

The chromalveolate hypothesis [1, 2] posits that four major lineages of protists – cryptophytes, haptophytes, heterokonts, and alveolates (dinoflagellates, apicomplexans, and ciliates) – form a monophyletic superassemblage (Chromalveolata) that is uniquely defined by a single secondary endosymbiosis between the putative common ancestor of this supergroup and an engulfed red alga. A key corollary of this hypothesis is that plastids were independently lost on numerous occasions within the chromalveolates, in the common ancestor of ciliates and from the basal lineages of the cryptophytes, heterokonts, dinoflagellates, and apicomplexans. The chromalveolate hypothesis was developed by Cavalier-Smith [2, 3] and is based largely on the number and types of membranes enclosing the plastid and the possession of chlorophyll c, as well as a desire to minimize the number of plastid symbioses.

Although several phylogenetic studies have found support for the monophyly of chromalveolate plastids [4-8] studies using cytosolic nuclear genes have not supported their monophyly. The chromalveolate hypothesis should at present be regarded as tenable but distinctly unproven (see ref. [9] for a strong challenge to this hypothesis). Although it is a reasonable possibility that host cells and nuclei of the chromalveolates do not form a monophyletic group, analyses using formally cytosolic nuclear genes adopted to function in the plastid [5, 7] provide strong evidence that the *plastids* in the chromalveolate lineages descended from a single red algal plastid.

Despite support for chromalveolate plastid monophyly the relationships between the various lineages remains uncertain. Cavalier-Smith [3] has argued, based on ultrastructural and pigment similarities, that haptophytes and heterokonts are sister lineages (forming the “chromobiotes”), that chromobiotes and cryptophytes together comprise the “chromophytes”, and thus that alveolates are sister to chromophytes. Although phylogenies based on concatenated plastid genes tend to group the haptophytes and heterokonts [6, 8], when dinoflagellates are included to represent alveolates [8], they tend to group specifically with either haptophytes or heterokonts, which is inconsistent with the chromobiote hypothesis [3]. Whereas cryptophytes are sister to the other chromalveolate lineages in the limited plastid analyses [6, 8], in analyses using formally cytosolic nuclear genes adopted to function in the plastid cryptophytes group with heterokonts with weak [7] to strong [5] support. Studies using six cytosolic nuclear genes group cryptophytes with haptophytes with weak support [10]. Although there is considerable support from multi-gene nuclear phylogenies for a close relationship of heterokonts and alveolates [10-13], in most cases no haptophytes or cryptophytes were included.

1. Keeling PJ: **Diversity and evolutionary history of plastids and their hosts.** *Am J Bot* 2004, **91:**1481-1493.
2. Cavalier-Smith T: **Principles of protein and lipid targeting in secondary symbiogenesis: Euglenoid, dinoflagellate, and sporozoan plastid origins and the eukaryote family tree.** *J Eukaryot Microbiol* 1999, **46:**347-366.
3. Cavalier-Smith T: **Genomic reduction and evolution of novel genetic membranes and protein-targeting machinery in eukaryote-eukaryote chimaeras (meta-algae).** *Philos Trans R Soc Lond B Biol Sci* 2003, **358:**109-133; discussion 133-104.

4. Fast NM, Kissinger JC, Roos DS, Keeling PJ: **Nuclear-encoded, plastid-targeted genes suggest a single common origin for apicomplexan and dinoflagellate plastids.** *Mol Biol Evol* 2001, **18:**418-426.
5. Harper JT, Keeling PJ: **Nucleus-encoded, plastid-targeted glyceraldehyde-3-phosphate dehydrogenase (GAPDH) indicates a single origin for chromalveolate plastids.** *Mol Biol Evol* 2003, **20:**1730-1735.
6. Yoon HS, Hackett JD, Pinto G, Bhattacharya D: **The single, ancient origin of chromist plastids.** *Proc Natl Acad Sci U S A* 2002, **99:**15507-15512.
7. Patron NJ, Rogers MB, Keeling PJ: **Gene replacement of fructose-1,6-bisphosphate aldolase supports the hypothesis of a single photosynthetic ancestor of chromalveolates.** *Eukaryot Cell* 2004, **3:**1169-1175.
8. Bachvaroff TR, Sanchez-Puerta MV, Delwiche CF: **Chlorophyll c-containing plastid relationships based on analyses of a multigene data set with all four chromalveolate lineages.** *Mol Biol Evol* 2005, **22:**1772-1782.
9. Bodyl A: **Do plastid-related characters support the chromalveolate hypothesis?** *J Phycol* 2005, **41:**712-719.
10. Harper JT, Waanders E, Keeling PJ: **On the monophyly of chromalveolates using a six-protein phylogeny of eukaryotes.** *Int J Syst Evol Microbiol* 2005, **55:**487-496.
11. Arisue N, Hashimoto T, Yoshikawa H, Nakamura Y, Nakamura G, Nakamura F, Yano TA, Hasegawa M: **Phylogenetic position of *Blastocystis hominis* and of stramenopiles inferred from multiple molecular sequence data.** *J Eukaryot Microbiol* 2002, **49:**42-53.
12. Baldauf SL, Roger AJ, Wenk-Siefert I, Doolittle WF: **A kingdom-level phylogeny of eukaryotes based on combined protein data.** *Science* 2000, **290:**972-977.
13. Simpson AG, Inagaki Y, Roger AJ: **Comprehensive multigene phylogenies of excavate protists reveal the evolutionary positions of "primitive" eukaryotes.** *Mol Biol Evol* 2006, **23:**615-625.
