# Supplementary material for: An exceptional horizontal gene transfer in plastids: gene replacement by a distant bacterial paralog and evidence that haptophyte and cryptophyte plastids are sisters
Source: BMC Biol. 2006 Sep 6;4:31. doi: 10.1186/1741-7007-4-31 (PMC1570145; doi:10.1186/1741-7007-4-31)
Supplement: Additional file 9 — Multigene chromist plastid phylogeny. Results of the phylogenetic placement analysis of the haptophyte Emiliania based on all plastid genes. [file 1741-7007-4-31-S9.pdf]

Multigene chromist plastid phylogeny

To evaluate the plastid based phylogenetic evidence for haptophyte/cryptophyte versus haptophyte/heterokont monophyly we performed multi-gene phylogenetic analyses of plastids using only genes and positions for which characters exist for a cryptophyte (*Guillardia*), a haptophyte (*Emiliania*), two heterokonts (*Odontella* and *Thalassiosira*), and two red algae (*Porphyra* and *Gracilaria*) (alveolates were excluded because their plastid genes are too few in number and too divergent in sequence). In agreement with previous multi-plastid-gene analyses [1, 2], this 97 gene, 21,659 amino acid concatenated dataset supports the sisterhood of heterokont and haptophyte plastids using the codon (BI = 1.0), amino acid (BI = 1.0, Additional File 10), and nucleotide (BI = 1.0) models in MrBayes, and using all codon positions (BP = 66%) or only 1st and 2nd positions (BP = 77%) in PAUP*. The branches leading to these three chromalveolate lineages are, however, very long and connected to each other by relatively short branches. This, combined with the very sparse taxon sampling yet large number of characters, sets up a worst case scenario for phylogeny reconstruction. Artefacts could readily occur due to statistical noise; slight biases in nucleotide, codon, and amino acid usage; and functional convergence and divergence.

We used the approximately unbiased test [3] to test the best topology against the *rpl36*-c inspired topology in which the haptophyte/cryptophyte monophyly is constrained. The topology of the remaining taxa did not change with this constraint (Additional File 10). Using the amino acid site log likelihoods calculated with codeml [4] the haptophyte/cryptophyte grouping was not rejected at the 5% level (p = 8.9%). Thus, even if one assumes that the underlying model of evolution is identical in the three chromalveolate lineages, the data do not confidently support one topology over the other. Beyond this it is easy to imagine that slight biases in the substitution process could, over such a long period of evolution, lead to artificial groupings. Such hypothetical biases could be global or gene specific. When we look at the phylogenetic signal on a per gene basis, half the genes (49.5%) favor the haptophyte/cryptophyte grouping (Additional File 10). Given these considerations, the molecular phylogenetic results are suspect at best. To resolve this issue using molecular phylogenetics, more taxa are needed to break up the long branches.

1. Yoon HS, Hackett JD, Pinto G, Bhattacharya D: **The single, ancient origin of chromist plastids.** *Proc Natl Acad Sci U S A* 2002, **99:**15507-15512.
2. Bachvaroff TR, Sanchez-Puerta MV, Delwiche CF: **Chlorophyll c-containing plastid relationships based on analyses of a multigene data set with all four chromalveolate lineages.** *Mol Biol Evol* 2005, **22:**1772-1782.
3. Shimodaira H, Hasegawa M: **CONSEL: for assessing the confidence of phylogenetic tree selection.** *Bioinformatics* 2001, **17:**1246-1247.
4. Yang Z: **PAML: a program package for phylogenetic analysis by maximum likelihood.** *Comput Appl Biosci* 1997, **13:**555-556.
